# BK virus salivary shedding and viremia in renal transplant recipients

**DOI:** 10.1590/1678-7757-2018-0435

**Published:** 2019-01-14

**Authors:** Dmitry José de Santana Sarmento, Michelle Palmieri, Gustavo Souza Galvão, Tânia Regina Tozetto-Mendoza, Cynthia Motta do Canto, Ligia Camera Pierrotti, Elias David-Neto, Fabiana Agena, Marina Gallottini, Claudio Sergio Pannuti, Maria Cristina Domingues Fink, Paulo Henrique Braz-Silva

**Affiliations:** 1 University of São Paulo, School of Dentistry, Stomatology Department, São Paulo, São Paulo, Brazil; 2 University of São Paulo, Institute of Tropical Medicine of São Paulo, Laboratory of Virology, São Paulo, São Paulo, Brazil; 3 University of São Paulo, Medical School, Hospital das Clínicas, Departament of Infectious and Parasitic Diseases, São Paulo, São Paulo, Brazil; 4 University of São Paulo, Medical School, Hospital das Clínicas, Serviço de Transplante Renal, São Paulo, São Paulo, Brazil

**Keywords:** Transplantation, BK virus, Saliva, Immunocompromised host, Polymerase chain reaction

## Abstract

**Objectives:**

This study aimed to verify the presence of polyomavirus BK (BKPyV) in the saliva of kidney transplant recipients and to correlate it with blood viremia.

**Material and Methods:**

We have conducted a cross-sectional study with a sample involving 126 renal transplant recipients. 126 samples of saliva and 52 samples of blood were collected from these patients. Detection and quantification of BKPyV were performed using a real-time PCR. To compare the presence of BKPyV in blood and saliva, the binomial proportion test was used. To verify associations between salivary shedding BKPyV and post-transplant periods (in months), the Mann-Whitney test was used. Spearman's correlation was used to correlate the viral load in the saliva with blood of kidney transplant recipients.

**Results:**

The mean age of the study group was 51.11±12.45 years old, and 69 participants (54.8%) were female, with a mean post-transplantation time of 4.80±6.04 months. BKPyV was quantified in several samples of saliva and blood, with medians of 1,108 cp/mL and 1,255 cp/mL, respectively. Only 16/52 (30.8%) participants presented BKPyV in blood, and 59/126 (46.8%) excreted the virus in saliva (p=0.004). BKPyV shedding was found in patients at a shorter post-transplantation period (3.86±5.25, p=0.100). A weak correlation was observed between viral quantification in saliva and blood (Spearman's correlation coefficient=0.193).

**Conclusion:**

The results of this study suggested that, although saliva excretes more BKPyV than blood, there is no reliable correlation between salivary shedding and blood viremia, showing two independent compartments of viral replication.

## Introduction

Currently, blood biochemical laboratory tests are the first choice for patient follow-up and for monitoring diseases. However, saliva has been suggested as an excellent material for diagnosis and monitoring, mainly because the collection of saliva is simple, painless, cheap and safe both for patients and the medical staff. In addition, saliva is a source of material that can be collected several times a day, without harming the patient^1^.

Polyomavirus BK (BKPyV) has a high prevalence of asymptomatic infection in the normal population and is able to cause kidney dysfunction in transplanted grafts via BK virus-associated nephritis, mainly because these patients are immunocompromised. Screening for BKPyV often reveals viruria and/or viremia, which can progress to active diseases^2^ ^–^ ^4^.

Kidney transplant recipients need special care and attention in the transplantation follow-up. Immunosuppression treatment is essential for the maintenance of kidney transplantation, but it negatively influences the balance between viral replication and cellular immune response. This leads to a potential risk of primary infections or opportunistic reactivations^4^ ^,^ ^5^. BKPyV causes 95% of nephropathy cases related to polyomavirus, with the remaining 5% being caused by JC virus. Nephropathy impairs graft function, causing its premature failure in 1% to 10% of the patients with kidney transplants^6^.

Since BKPyV remains latent in renal tissue, kidney transplant recipients are monitored with great interest for detection of viruses in order to prevent overt clinical disease. Early diagnosis and immunological recovery are essential to prevent transplant rejection in these patients. Non-invasive screening can facilitate the detection of new cases and monitor previously known cases^2^ ^,^ ^6^ ^–^ ^8^. Thus, it is important to know the profile of BKPyV oral shedding in these patients and to understand the clinical importance of this excretion in saliva for them. The objective of this research was to verify the presence of BKPyV in the saliva of kidney transplant recipients and to correlate it with blood viremia.

## Material and methods

### Design and study population

A cross-sectional study was conducted in which the subjects were part of a convenience sample of the Kidney Transplant Unit of the University of São Paulo, São Paulo/SP, Brazil. This study was approved by the local Research Ethics Committee according to protocol number 0234/10 with all the participants signing an informed consent form.

Demographic data were collected, including information on gender, age and post-transplant period at the time of data collection. The inclusion criteria were being over 18 years old, having had kidney transplant only regardless of the post-transplantation time, whereas the exclusion criteria were using antiviral drugs in the past three months before sample collection, being HIV-positive or having undergone multi-visceral transplant.

Study population involved 126 kidney transplant recipients. 126 samples of saliva were collected, and, from these same patients, 52 blood samples were also collected (only from those who consented to blood collection). All samples were collected at the same appointment, randomly. Approximately 3 mL of non-stimulated saliva were collected into a 50 mL Falcon tube by using drainage method, with the first-minute collection being discarded. Five milliliters of blood were collected and put into a tube containing coagulation factor, and aliquots of blood sample were separated to analyze BKPyV. All samples were stored at −80°C.

### DNA extraction and quantification by using quantitative real-time PCR

Detection and quantification of BKPyV were performed by using a real-time PCR, and laboratory procedures were conducted at the Laboratory of Virology of the University of São Paulo Institute of Tropical Medicine (IMT-USP)^2^ ^,^ ^3^. DNA extraction was performed using the QIAamp DNA extraction QIAGEN kit, according to protocol for RT-qPCR in which primers and specific probes are used to detect the gene encoding the Ag-T protein of the BKPyV, resulting in a 80-pb fragment (Forward: 5-GAAACTGAAGACTCTGGACATGGA-3; Reverse: 5-GGCTGAAGTATCTGAGACTTGGG-3; BKPyV probe: CAAGCACTGAATCCCAATCACAATGCTC)^9^.

The reaction probes were labeled with FAM (6-carboxyfluorescein) as a reporter dye at the 5′ end, and with TAMRA (6-carboxy-tetramethylrhodamine) as a quencher at the 3′ end. The final reaction was prepared with 12.5 μl of TaqMan Universal PCR master mix (2x) (Applied Biosystems®, Carlsbad, CA, USA), 0.5 μl of each primer (10 μm), 0.5 μl of probe (5 μm) and 6 μl of DEPC water. The reaction parameters were 2 minutes at 50°C and 10 minutes at 95°C, followed by 45 cycles (15 seconds at 95°C and 1 minute at 60°C) by using an ABI 7300 PCR machine (Applied Biosystems®, Carlsbad, CA, USA). A plasmid pattern containing the large T antigen encoding region was used as a control to determine the number of copies per milliliter in the final reaction. The protocol employed has a high analytical sensitivity, that is, a detection limit of 1000 copies *per* milliliter (cp/mL) of blood.

### Statistical analyses

To compare the presence of BKPyV in blood and saliva, the binomial proportion test was used. The variables “post-transplantation time after kidney transplantation” and “quantification of BKPyV in saliva and blood” did not present normal distribution (Kolmogorov-Smirnov, p<0,001). To verify the associations between salivary shedding BKPyV and post-transplant periods (in months), the Mann-Whitney test was used. Spearman's correlation was used to correlate the viral load in the saliva and blood of kidney transplant recipients. The resulting data were analyzed through the SPSS 17.0 software (SPSS Inc., Chicago, IL, USA). All tests were performed considering a significance level of 0.05.

## Results

The mean age of the participants was 51.11±12.45 years old, of whom 69 (54.8%) were male and 57 (45.2%) were female. The mean post-transplantation time after kidney transplantation was 4.80±6.04 months. BKPyV was quantified in 126 samples of saliva, with a mean of 4,761±11,105cp/mL (minimum= 63; maximum= 55,241, median= 1,108), and in 52 blood samples, with a mean of 93,604±364,227cp/mL (minimum= 80; maximum= 1,459,398, median= 1,255), with the latter showing higher viral quantification. Only 16/52 (30.8%) participants presented BKPyV in blood, whereas 59/126 (46.8%) excreted the virus in saliva (*p*=0.004) (Table 1). BKPyV shedding was found in patients with shorter post-transplantation period (3.86±5.25, *p*=0.103) (Table 2).

**Table 1:** Detection and prevalence of BKPyV in blood and saliva samples

BKPyV/SAMPLE	n	%	Prevalence	p
BLOOD				
Positive Negative	16 36	30.8 69.2	30.8%
TOTAL1	52	100		
SALIVA				0.004*
Positive	59 67	46.8 53.2	46.8%
Negative				
TOTAL	126	100		

^1^Blood sample was collected from 52 patients

* Binomial proportion test, statistically significant results

**Table 2:** Relation between salivary shedding of BKPyV and post-transplantation time

	n	%	Post-transplantation time (months) mean+SD	p
		Positive cases		
BKPyV in saliva	59	47.2	3.86+5.25	0.103^1^
	Negative cases			
	66	52.8	5.64+6.60	

SD: standard deviation

1 Mann-Whitney

* One patient did not present any information on post-transplantation time

Fifty-two patients had paired samples of blood and saliva, and of these, only 11 (21.1%) had viremia and salivary shedding of BKPyV at the same time. Of these 52 individuals, the mean viral load in saliva was 1,079±2,916 cp/mL, and in blood it was 28,801±202,289. The viral load in saliva was correlated with the blood from these samples, and a weak correlation was observed between salivary shedding and blood viremia of BKPyV (Spearman's correlation=0.193, p=0.170).

## Discussion

BKPyV frequently reactivates in kidney transplant recipients due to immunosuppression. In urine, the BKPyV had a prevalence between 20.4% and 40%^7^ ^,^ ^8^ ^,^ ^10^ ^–^ ^13^, with BKPyV viruria being detected in 4-5% of the kidney transplant recipients. PCR monitoring of BKPyV with urine and blood samples is a rapid and beneficial method to prevent renal complications during long-term care of kidney transplant recipients^10^ ^,^ ^13^ ^,^ ^14^.

However, oral fluids (e.g. saliva) have exhibited high prevalence of BKPyV, being also efficiently detected compared to urine and blood^2^. Furthermore, because saliva contains several biological markers which can also be detected in urine or blood tests, salivary fluids can be used to detect and monitor several pathologies with the same effectiveness^1^ ^,^ ^15^.

Although saliva is a potential material for diagnosis, it is also an important mean of contamination, which should be considered in kidney transplantation patients. Studies have shown that the prevalence of BKPyV in the saliva of these patients reaches 91.7%^2^, and it can be detected in the saliva of HIV-positive patients as well^16^. Oral fluids exhibit high prevalence of BKPyV and are equally efficient compared to urine and blood. When oral fluids are used in screening assays to detect polyomaviruses, the results are highly positive and are especially indicated for patients who are unable to urinate. This positivity in oral fluids occurs even when the patient has negative viremia, which indicates that saliva can be a means for early detection of BKPyV^2^ ^,^ ^3^.

In our study, 46.8% of the participants excreted the virus in their oral fluids, which is a lower percentage than that observed in literature. Diseases related to BKPyV may be associated with a specific immune deficiency as a result of the use of immunosuppressive drugs^3^ ^,^ ^17^.

The BKPyV was detected in oral fluids and infections, with its replication occurring *in vitro* in salivary gland cells, which justifies the BKPyV presence in the saliva^18^. These results support ours, especially when the presence of BKPyV in the saliva of kidney transplant recipients is significantly greater than in their blood.

In our study, the viral loads were higher in blood (93,604±364,227cp/mL) than in saliva (4,761±11,105cp/mL). Despite the presence of outliers, we believe there are strong indications that there are differences in quantification between saliva and blood, meaning that other studies should be conducted to confirm this hypothesis. An increased BKPyV replication occurs in immunosuppressed patients, resulting in the destruction of infected uroepithelial cells and increased inflammatory immune cells^19^. In renal transplant recipients, this destruction affects 5-8% of the kidney transplantations and is called BKPyV-associated nephropathy, which may result in graft-failure in the transplanted recipients^18^ ^,^ ^20^ ^,^ ^21^. In this sense, saliva could be useful to detect viral levels predisposing to the development of this type of nephropathy. However, our results demonstrate that saliva is not reliable for this evaluation. Therefore, clinical and prospective studies are necessary to confirm this hypothesis.

A weak correlation between viral quantification in saliva and blood was observed in our study, showing two different and independent compartments of BKPyV replication. A recent study of polyomaviruses miRNA expression in saliva have shown that the oral cavity is a region of persistent infection of BKPyV, more frequently than plasma^22^. The fact that there was no concordance implies that replacing tests traditionally used for urine and blood screening is not recommended. Nevertheless, the use of oral fluids concomitant with other fluids enhances positive screening and is useful as a complementary test to detect polyomaviruses^2^. Although this study suggests that salivary shedding of BKPyV does not correlate with the active disease, one of its limitations was that we did not seek to associate the active disease with the salivary shedding of BKPyV. Thus, clinical studies with similar methods are suggested in kidney transplant patients with an active disease.

BKPyV shedding was found in patients with shorter post-transplantation period, but with no statistical significance. However, this result is consistent with the concept that infections in kidney transplant patients are more common in the first months after transplantation, when they have maximum immunosuppression^5^ ^,^ ^23^.

Mouth-washing with Listerine® has been used as an alternative method to collect saliva, being effective in viral preservation for analysis by real-time PCR. One study showed high frequency of detection and quantification of BKPyV with mouth-washing (23 of 46 subjects), suggesting that such a method is also a good alternative for detecting and monitoring this virus^2^. We used the method of non-stimulated saliva collection, and the results were similar to those of the mouth-wash collection with Listerine® regarding the analysis through real-time PCR. These results suggest that the form of saliva collection has no influence on the laboratory analysis for BKPyV.

## Conclusion

In conclusion, BKPyV was more common in saliva than in blood, especially in recently transplanted patients. A weak correlation between salivary shedding and blood viremia was observed. Therefore, this study suggested that, although saliva excretes more BKPyV than blood, there is no reliable correlation between salivary shedding and blood viremia, showing two independent compartments of viral replication.
